# Mind-Object Identity: A Solution to the Hard Problem

**DOI:** 10.3389/fpsyg.2019.00063

**Published:** 2019-02-05

**Authors:** Riccardo Manzotti

**Affiliations:** Department of Business, Law, Economics and Consumer Behaviour, Università IULM, Milan, Italy

**Keywords:** consciousness, hard problem of consciousness, naturalism, hallucination, argument from illusion, physicalism, philosophy of mind

## Abstract

Here I present a mind-object identity theory based on a straightforward hypothesis: One's experience of an object is identical with the object itself. To defend this hypothesis, I will reconsider the notion of a physical object in terms of relative and actual properties. To address cases of misperception such as dreams and hallucinations, I will also reconsider the notion of present in relative terms. Both the object and the present are recast as object-relative.

## Introduction

I present a mind-object identity theory based on a straightforward hypothesis: *One's experience of an object is identical with the object itself*. To defend this hypothesis, I will reconsider the notion of a physical object in terms of relative and actual properties, and in doing so, I will support a form of naturalism.

At the outset, I'd like to stress that the proposed view is neither a form of panpsychism nor a variant of neutral monism; moreover, it does not require any emergence. The goal is showing that if nature is reconceived according to a relative notion of physical objects, consciousness will fit seamlessly into what we call the physical world.

“Physicalism” is a notoriously subtle and elusive view, of which there are several variants. I agree that it is impossible to define a priori the nature of the physical (Russell, 1927; Strawson, 2017), therefore the term will be used in the context of being part of the domain of which pebbles, molecules, and radio waves are relatively uncontroversial examples. Such an approach is routinely adopted by physics—e.g., if something behaves like other physical stuff, it is taken to be part of the physical world. As a result, in this paper, anything that is located in space-time, that is causally relevant (more on this soon) and that is made of matter/energy will be taken to be physical. Thus, a packet of energy or a pebble are suitable example. Since the focus is on our experience in everyday standard situations, I will focus mostly on everyday familiar objects such as tables, rooms, clouds, and also phenomena such as rainbows, flashes, constellations and pieces of music. Many philosophers have done the same (Kim, 1998; Merricks, 2001; Brewer, 2006).

The goal of the paper is to show that, by revisiting the nature of the physical world in terms of relative properties, it is possible to reveal a *mind-object* identity that, in contrast with the traditional *mind-body* identity, maintains that while consciousness is located in the physical world, it is not identical to brain processes but rather to external objects. In brief, the proposal is that, whenever a subject S experiences an object O, S's experience is nothing but O itself. O is a relative object where *relative* reads as in “relative velocity” rather than as in “relative to a subject.”

Such a proposal, of course, does not entail any form of panpsychism or idealism. In other words, if experience is identical to an object, it does not entail that objects are identical with a mental world. Instead, identity moves in an arrow from experience to the physical world. Consider the traditional mind-brain identity theory, such as Smart's version where he suggests that “sensations may be just brain processes” (Smart, 1959 p. 141). This hypothesis, where sensations may be nothing more than brain processes, does not entail that brain processes were sensations (as in Russell's neural monism). Likewise, I propose that sensations/experience may simply be properties of physical objects in the external world.

I will organize the paper as follows. First, I will outline the main idea in the case of successful standard perception, which is perhaps the most favorable case. Then I will list three key objections: subjective variability, alleged irreducible mental features, and the argument from illusion in all its variants. To address such objections, I will delve into an articulate account of the proposed building blocks of nature—i.e., the relative object—and I will articulate the role of the body as a condition of existence for the object itself. With these two key notions, I will carefully address the three objections plus a hoard of other recurring worries.

## Mind-object identity

Does consciousness fit into the physical world? Many philosophers and scientists believe that it doesn't unless extraordinary hypotheses—such as emergent properties, integrated information, dual aspect information, and the like—are deployed (Koch and Crick, 2001; Koch, 2009; Eagleman, 2011; Tegmark, 2014; Tononi et al., 2016). According to many of the most influential philosophers of mind, consciousness is akin to Monty Python's Spanish Inquisition, namely it is totally unexpected (Nagel, 1974; Shoemaker, 1982; Chalmers, 1996). Of course, a widespread belief is that consciousness is somewhat located in the brain (the alleged physical substrate of consciousness). Very often such a belief implies that consciousness either supervenes on phenomena taking place in the brain or that consciousness is identical with the brain's processes as the mind-brain identity theorists proposed originally (Smart, 1959; Armstrong, 1968).

In fact, if successful from a scientific perspective, an identity theory would be a welcomed solution because it would locate consciousness in the physical world. Unfortunately and differently from a large body of literature (Kripke, 1972; Levine, 1983; Jackson, 1986; Chalmers, 1996), which has contended that the purported identity is conceptually flawed—I will maintain that the biggest shortcoming of identity theories is of empirical nature: brain processes do not have the properties that consciousness has. While this incongruence may seem a trivial point, it remains, I believe, a key obstacle that identity theories must face. No matter how many scholars have claimed that mental states and brain states are the same, they do not seem so. Brain states and consciousness simply do not have any property in common. In response to such an empirical fact, scientists and philosophers alike have deployed form of denial—e.g., consciousness is there, but it cannot be seen, or consciousness is identical with brain processes, but it appears different (for instance by appealing to an alleged difference between first person and third person access, emergence, dual aspect of information, and other esoteric excuses). Yet, notwithstanding the failure of mind-brain identity, the notion of identity is not necessarily confined to the brain. So there is still hope that it can be resurrected. Consequently, it makes sense to consider whether consciousness, being real, might be identical to an *x*, where *x* must be physical but not necessarily in the brain. The form of the solution proposed by identity theorists might have been correct: conscious experience is *x* (*x* being a physical phenomenon). However, they might have chosen the wrong *x*. In fact, as argued above, the hypothesis that *x* is a brain process has proven to be empirically wrong. Then, what else might *x* be? Remarkably, one candidate has gone unnoticed, a candidate that has been *hidden in plain sight*: the external object.

Whenever standard perception takes place, along with one's body and one's brain, there is also the external object. Could the object be the thing that is identical to one's experience? Strange as it may seem, this is the case I will defend here. When it comes to cases of successful standard perception, the proposal is that *x* is the external object. In short

S's experience of O is O itself

Where O is the external physical object—no Berkeley's ideas are advocated. An example will help. Consider an ordinary (and favorable) case of successful standard perception: Jacqueline perceives an apple and there is an apple. From a physical perspective, there are two physical objects interacting together—the apple and Jacqueline's body. Next, of course, there is the issue of Jacqueline's conscious experience of the apple. *What and where is such an experience?* If one is a physicalist, there must be something that is one and the same as her experience, and such a something must be physical. In addition, for identity to work, there must be something with the properties of Jacqueline's experience. Yet what? Traditional mind-brain identity theories have suggested looking for it into one's brain. Yet, nothing inside Jacqueline's brain has the properties that Jacqueline finds in her experience. Jacqueline experiences a red, round, and shiny apple, but nothing inside her brain is red, round, or shiny. How could a brain process be identical with Jacqueline's experience of the apple? Simply put, it cannot. We can thus consider another candidate, which is the only thing around that has the properties of Jacqueline's experience of the apple: the apple itself. The undoubtedly quite radical hypothesis is that Jacqueline's experience of the apple is nothing but the apple that exists relative to Jacqueline's body (more on the relative nature of the apple soon). The solution simplifies our understanding of the case. Two objects are there: her body (or her brain) and the apple. One of them is Jacqueline's experience. Only it is not her body but the apple. In this way there are no more metaphysical mysteries. What's going on, though not easy to know in detail, is purely physical. Everything is physical.

This solution entails neither panpsychism nor idealism but it defends a reduction of experience to the properties of physical objects that exist relative to one's body. If correct, there is no need to introduce any problematic notions. In the end, there is only the physical world and its properties. The proposed solution suggests how to locate those properties that tradition has placed in a mental world and mistakenly considered to be metaphysically different.

The proposal is straightforward: Jacqueline's experience of the apple is nothing but the apple itself—*one's experience of an object is the object one experiences*. Such a hypothesis is the core of the Spread Mind theory (Manzotti, 2016, 2017b,c). One's experience is not located inside the brain, it is the very object one experiences. Consciousness is literally out of the head (Parks, 2018).

So far, Jacqueline's experience of the apple—which is red, round, and shiny—has been puzzling because, to the best of our knowledge, in the physical world everything is just what it is. The apple is the apple. Jacqueline's brain is Jacqueline's brain. The table is the table. The Sun is the Sun. So, if consciousness were a process in the brain, it would access the properties of the apple without having them. This would require phenomenal metaphysical superpowers (pun intended). For a traditional identity theorist, the brain is forced to do something that in nature nothing else does—namely, reaching out and becoming something else aside from itself. If Jacqueline's experience were identical with her neural activity, how could such an activity partake of the properties of the apple? Contrary to current views, there is only one thing applish enough to be Jacks' experience: the apple.

The hypothesis that our experience of an object is nothing but the object as it exists relative to our body represents a huge conceptual leap. Yet, why not? After all, the suggested mind-object identity is strictly physicalist. The form of the explanation is the same of the traditional mind-brain identity theories. While they proposed to consider brain processes, I propose to consider external objects. Such a view offers unprecedented ontological parsimony. Not only does it get rid of mental properties, it also sets all subject-object relationships aside—e.g., intentionality, perspectivalness, and phenomenal character.

A caveat. The theory being proposed is neither a new version of panpsychism nor a rendition of neutral monism. Rather it is a strong form of physicalism and realism. Moreover, the proposed theory does not require the addition of any phenomenal quality to the physical world. I want to be very clear on this point. The theory does not claim that the external object instantiates phenomenal properties. Rather, the external object is the experience. The physical world is assumed to be sufficient to host the thing that we have been referring to as consciousness. The reason why experience and world have been kept apart—I will argue—does not lie in their ontological incongruity, but in a series of questionable premises.

The suggested conceptual shift, while theoretically momentous, does not contradict any empirical evidence. Moreover, it goes further than other attempts that have taken into consideration the dependence between one's experience and the external word such as various forms of externalism, enactivism, extended cognition, and radical embodiment (Clark and Chalmers, 1998; O'Regan and Noë, 2001; Chemero, 2009; Noë, 2009; Hutto and Miyn, 2017). While these approaches, by and large, maintain that experience is constituted by the relation between the body and the external objects, my proposal is much more radical—experience is identical with the external objects themselves.

## Three key objections

Thus, far I have approached the mind-object identity in the most favorable case of successful standard perception: S perceives O and O is there. Now we can turn to more challenging and interesting cases. In particular, we must address considerable objections from subjective variability, from alleged irreducible properties of the mental, and from the argument from illusion (hallucinations, dreams, illusion, mental imagery, and memory).

*Subjective variability* refers to the easily observed fact that an object appears differently depending on the subject. The same apple may appear bigger to a child or smaller to an adult. The temperature in the room may seem cold if one has a fever and warm if one has finished an intense running session. If experience is the external object and the external object is the same for everyone, why do we not all experience the same object? Is this proof that we experience subjective objects? Is this evidence that the phenomenal character of our experience is the outcome of some internal processing? I argue it is not. It is only the consequence of the relative-yet-utterly-physical nature of objects and their properties.

Secondly, the mental is *allegedly characterized by irreducible features*—such as Nagel's well-known question about what it is like to be something (Nagel, 1974). Yet, is the “what it is like” really different from the “it” itself? What evidence do we have that the phenomenal is not the physical? Are there any irreducible features of the mental? An identity theory must account for all the properties that we find in our experience and must be able to locate them. For the sake of simplicity, I will focus on two of the main allegedly irreducible qualities of the mental: intentionality and phenomenal character. I will argue that they are either philosophical inventions (as intentionality) or are one and the same with physical properties (as phenomenal character) which are indeed expressed by physical objects without any need for adding extra ontological levels (as panpsychists, neutral monists, and dualists have often done).

Finally, the well-known *argument from illusion* will be examined through dreams and hallucinations. Occasionally, S perceives O and there is no O. If one experiences O, should O not be there? How can this view, based on an utmost realism about nature, counter such arguments?

Fortunately, and perhaps surprisingly, the same line of defense will suffice against all such objections—a fully-physicalist revision of the notion of an object in terms of relative existence. In fact, to put forward a mind-object identity theory, one needs to ground the theory on a reasonably clear notion of what an object is. A physicalist theory of consciousness is first and foremost a theory of existence (of objects). Only in this way will we be able to check the properties of nature against those of our experience and determine whether they are the same.

## The relative object

The subject-object dichotomy might have stemmed from an oversimplistic notion of the physical world that has encouraged scholars to add the level of the mental—fittingly nicknamed the “dustbin model of the mind” by Sydney Shoemaker (Shoemaker, 1996). If this is the case, a key step will consist in revisiting the physical world and its most familiar constituent—the object. The commonsensical notion of a physical object is that of an individual with properties that are independent of surrounding circumstances and that can be defined in a causally inert and atemporal way. For instance, typically a canvas is taken to have a given color and size independent of other circumstances. This is the idea behind the notion of objective measurement: an object is thus and so due to its objective properties that do not depend on the way they are measured. The canvas is, let's say, one square meter large and antique white in color independent of either ruler or viewer. Likewise, an apple is taken to be red, round, and shiny independent of other circumstances. At the very least the fruit is taken to have the disposition of being red under natural light in normal conditions. In general, properties are taken to be instantiated by each object without recourse to the rest of the universe. Yet this notion, as familiar as it might sound, runs afoul of both quantum mechanics and relativity theory, where properties are understood to be relative and largely dependent on the physical systems with which they interact. Remarkably, the commonsensical notion of object runs afoul also of Galilean relativity where certain properties—e.g., velocity—were revealed to be intrinsically relative. Finally, in philosophy, such a familiar notion is challenged by both the Eleatic principle and Alexander's dictum (Alexander, 1920; van Inwagen, 1990; Kim, 1998; Merricks, 2001), which state that a property exists only if it is causally active and thus only when it has the opportunity to produce effects.

To overcome the abovementioned downsides, physical objects will be recast as relative objects following Galilean relativity and/or the Eleatic Principle, as is the case with relative velocity. By and large, I propose to generalize the idea of relative existence to *all* physical properties (and thus to objects themselves).

The key point is to acknowledge the relative and actual nature of physical objects and of their properties. Here I take *relative* to roughly mean that each property of a physical object (at least those that partake our experience) depends on other objects and is therefore intrinsically relative (as expressed by Galilean relativity). By *actual* I mean that each property of a physical object is embedded and defined by the occurrence of an actual physical process that is located in space and time (as suggested by the Eleatic principle).

Remarkably, all physical properties we are familiar with exist only relative to other objects. In our own case, the object they are relative to is our own body and, more specifically, our sense organs and brains. Note that this fact does not entail any special metaphysical status for human bodies—i.e., a body is just yet another object.

The kind of relativity advocated here is object-object relativity. Consider the case of velocity. Ever since Galileo, it has been well-known that velocity is relative—i.e., there is no such a thing as absolute velocity. However, this physical fact does not entail that velocity is either subjective or arbitrary, but only that velocity is relative. Relative velocity is a well-defined quantity. In other words, velocity is not *subject-dependent* but *object-dependent*.

Distinguishing object-dependence from subject-dependence is paramount. In fact, in the philosophy of mind, relativism and relationalism have often been associated with mind-dependence and thus with subjectivity. This is not the case. The simple case of velocity suggests a different (and simpler) picture. Velocity is inherently relative, and it is relative to other objects (or their reference frames).

Object relativity addresses the traditional question: “Would a tree falling in a forest with no observers make any sound?” as we would with the question “would a particle moving in an empty space with no other particle have a velocity?” In both cases, the reply is negative—not because the sound is taken to be a mental entity created by a subject, but rather because the sound is taken to be a relative physical entity coming into existence because of the causal circumstance offered by a human body, which is just yet another object. The same rationale holds for traditional relative properties such as velocity. If an object were utterly isolated, would it have any velocity? Of course not. The question would be meaningless. An entirely isolated object is neither in movement nor at rest. Likewise, without the body, the opportunity for the existence of the sound is lost. The tree will fall, and the air pressure waves will scatter, however their chance to create a sound will be lost due to the absence of a human body for the sound to occur. The sound is neither a process inside the human body nor a mental entity inside the mind. In this account, the sound is the physical cause of an effect on a human body. Without the body such an effect will not occur and thus no cause will either.

The traditional notion of subjectivity, as something *whose existence is relative to a subject*, is thus substituted by that of object-relativity, as something *whose existence is relative to another object*, which is, in our case, the human body. The latter notion, which is compatible with Galilean relativity, is enough to cope with the fundamental features we have always associated with phenomenal experience—or so I will argue later.

Why has the relative nature of the physical world—object relativity—so often been dismissed? The likely reason is that, in practice, the stipulation of a conventional reference frame allows people to omit the otherwise necessary yet verbose mention of a conventional reference frame. When we say that a car is traveling at 50 mph, we omit saying that the car is traveling at 50 mph *relative to the ground*. The ground is the conventional reference frame. Similarly, when we say that a copper wire has a voltage of 50 Volt, we omit saying that it has a voltage of 50 Volt *relative to the ground*. And so forth. So, people tend to forget that these properties exist relative an implicit conventional reference frame. Of course, the adoption of an implicit conventional reference frame does not make such properties any less relative. As we will see later, this is also the reason why we so easily dismiss the causal role of our body in shaping our world. Since one's body is always present as the conventional causal reference frame of one's world, one easily forgets its key role.

The layman's standard view takes the world to be made of physical properties (objective and absolute) and mental properties (subjective and arbitrary). In contrast, I suggest that all properties are relative and that the former are simply conventionally chosen relative properties. The so-called objective properties are no longer the real properties the world is made of, they are only canonical properties that have been historically selected, just like the Greenwich time zone, which is no more real and objective than, say, Brisbane's time zone.

Importantly, the object is not only relative, it is also *actual*. The objects and their physical properties are always spatiotemporally located and causally efficacious. While one could argue that there may be entities that do not produce any effect whatsoever, these entities would be, by definition, epiphenomenal and outside the scope both of our experience and of scientific observation. An entity that does not produce any effect can neither be observed nor acted upon, and, thus, it is, to all extents, causally extraneous to our world. So, even if such entities existed, they would not be the entities our world is made of. In fact, in physics all properties manifest causally themselves by means of their effects. Such effects bring the physical properties (and thus objects which are bundles of properties) into existence and fix the object in a precise spatiotemporal location. The actual nature of the object is a nomological consequence of standard physics—it follows from the upper speed limit of all physical processes. Consequently, relative properties need time to be pulled into existence and thus are spread over time spans of arbitrary length. The bottom line is that all known physical properties in our life are both *relative* and *actual*.

The proposed revision is neither theoretically demanding nor scientifically challenging. It does not conflict with physics. It is consistent with both Galilean relativity and Einstein's notion of speed limit. Finally, this revision does not endorse anything like backward causation or processes snapping back in time. Nothing goes back in time. While relative objects are spatiotemporally located, because the object with its relative properties remains undefined until such properties are fixed by the conclusion of a process, the object is ontologically mature, so to speak, only after its (relative) properties get fixed by ensuing events. The flow of time moves only forward.

In sum, I suggest that all the properties we are familiar with are physical properties that exist relative to other objects. Everything is object relative. Relative objects allow us to outline an utterly physicalist view of both objects and experience—the two being the same. The world is made of objects. Each object exists because its properties take place thanks to some further object. In such an object-only physical world, everything is physical, everything is an object. Each object is, at the same time, a cause and an effect. It exists because it causes effects on other objects and it allows other objects to exist by their acting upon it. In such a world, everything is identical with itself and there is no need for additional ontological levels, emergent properties, mental states, phenomenal characters, or ontologically equivocal relations. Our conscious experience is where the world is. It is that world. On such a view, the ontological separation between subjects and objects disappears—subjects are only those particular objects we are identical with, but no metaphysical gap divides them.

## The Role of the Body

What is the role of our body then? The body is just yet another object. However, in our case, it is an important object because it is the *causal reference frame* relative to which all the objects in one's world take place. The body is analogous to a conventional reference frame. The body is always there, and thus it can be ignored. Yet, our body, for each of us, is paramount because it is the object relative to which everything, we are identical with, exists. If one's body is destroyed, one's world is destroyed too.

Consider the analogy of a system composed by a lake and a dam. The water flows in a river. Because of the dam, a lake fills up. Before the dam was built, no lake filled the valley. However, because of the dam, the water formed a lake. The water is not just the lake and the lake is not just water. The lake exists because of the dam. The dam was built because of the river and the possibility of creating a lake. The dam does not create the lake out of thin air, though. If no dam had been erected, no lake would exist. Crucially, the lake is not an emergent property of the dam. If there were no rain, a one-mile-high dam would not create a lake autonomously. Rather, the lake takes place because of the dam and the river, and because of several other conditions. Yet, in that valley, the dam has a key role in bringing the lake into existence. To extend the analogy, the lake is the object. The river is the incoming flow of events. The terrain is the environment. The water is the stuff the world is made of before one experiences it. The dam is one's brain and body. Where is one's experience? It is the lake. Where is the object? It, too, is the lake. The key idea is that the existence of the object is dependent on the proper causal circumstances. My conscious mind is the lake. My body is the dam. My experiences exist thanks to the body, and yet they are not the body. My experiences are nothing but the properties of the physical objects that take place relative to my body. Please note that this account brings together the relative and actual nature of external properties. Relative because they are what they are in relation to the body they interact with. Actual because they exist thanks to the actual occurrence of an effect in another body.

The body shapes the world that surrounds us, which is a relative world. Consider a familiar property such as color. A canvas is white. By “white” we usually mean that the canvas appears white to a standard trichromat under direct sunlight relative. If we think carefully about this definition, it is quite clear that even once we have satisfied the environmental conditions (direct sunlight) the color of the canvas is still dependent on the observer. What if the observer is not a standard trichromat? What if an observer is red blind? Would the canvas still be white? Fortunately we can do a simple and easily replicable experiment whose results are very well-known since Helmholtz's pioneering work (von Helmholtz, 1924; Brindley, 1963; Jones, 1972; Hurvich, 1981; Pridmore, 1999, 2008; Manzotti, 2017a): i.e., color afterimage. If one observes a saturated red patch for 30 continuous seconds one will become partially red blind. As a result, if one immediately stares at a gray patch afterwards, one will see a green-blue patch. The explanation is simple. Trichromatic vision is based on three main color components: red, green and blue, which is of course only a crude approximation of the L, M, S curves of spectral absorption (Stockman and Brainard, 2010). Due to the prolonged observation of a red patch, one's receptors of red adapt and therefore lose some of their sensibility. In short, one will become slightly red blind. Thus, one will see the chromatic leftovers—namely the green and blue components of light whose combination is cyan. In other words, if one is a fatigued trichromat whose red receptors have temporarily become partially blind, the color of the canvas will no longer be white but cyan. It is customary to account for these circumstances by appealing to an illusory mental cyan, but is it necessary? After all, why should the standard trichromat be any better than the fatigued trichromat? They are just two among infinite possible combinations of receptors. The standard case does not offer any privileged access to the *true* color of the canvas. The first case is conventionally representative of the real color of the canvas because it is more common and it is the one that corresponds to a healthy average human being. The color is relative to the physical system with which a surface interacts. So, the same canvas will be white *relative to* a standard trichromat; cyanish *relative to* a trichromat who is red-fatigued; magentish *relative to* a trichromat who is green-fatigued, and so forth. There is no absolute color, there are only colors *relative to other objects*—in this case, the visual systems of the human beings involved. Thus, color is analogous to velocity and white is analogous to the velocity relative to the ground. The alleged objective properties are nothing but conventionally chosen relative properties. The standard trichromat and the ground are conventional reference frames. “Relative” does not mean being relative to a subject (being subjective), but rather being relative to another object. *Object*-relativity does not imply *subject*-relativity nor subjectivity.

Consider now a white patch on a computer screen. From a comfortable distance, the surface of the display appears to be white. Now, if you get much closer, you will distinguish the physical structure of the display. At such a close distance you will resolve an array of the red, green, and blue light-emitting diodes. The patch will no longer appear white, but rather as a mosaic of red, green, and blue points. Is the patch white or is it a mosaic of colored lights? There is not a unique answer. The screen is both solid white *relative to* a human visual system at, say, 50 cm and a colored grid *relative to* a human visual system at, say, 1 cm. If the interacting system was a visual system with super visual acuity even at 50 cm, the screen would be a mosaic of colored diodes. In fact, phone and tablet screens are optimized to exist relative to a standard user at a standard distance. The color of the screen (being white or colored) does not depend on the object alone, but on the physical system the screen is relative to.

All physical properties, like velocity and color, are relative to other objects. Here I cannot address all the properties we experience, yet I contend that it is possible to make an equally convincing case for all of them. In short, each physical object we experience is a bundle of relative properties that exist thanks to the interaction with our body—i.e., it is an object relative to our body. So, the canvas is not white in any absolute sense; it is white relative to a standard trichromat and cyanish relative to a red-fatigued trichromat. The existence of the world we live in is relative to our body; it is made of those objects that exist relative to our body, which is our natural causal reference frame. Like all conventional reference frames, it tends to be forgotten, since it represents the ever present condition of existence of everything our world is made of. The role of our body in shaping our world is so largely invisible unless something (often bad) happens. For example, in the case of hemineglect, the world in which one lives is dramatically reduced. The damage to a cerebral hemisphere destroys the causal circumstances that allow the existence of half of one's world. The subjects affected by such a condition behave and feel as though half of their world had disappeared. The world we live in is a sort of negative projection of our body's sensorial apparatus, which is the collection of those organs that are causally coupled with the external world. “Sensorial apparatus” is a sort of misnomer because our organs do not sense anything. Sense organs contribute to the causal circumstances that allow the existence of those objects our experiences are identical with. The layman's notion that we have an experience of the world thanks to our body corresponds to the fact that the world relative to our body comes into existence thanks to our body. Our body, though, does not sense anything.

## Subjective Variability

What about subjective variability? Why do different subjects experience the same object differently? If our experience was the object itself, why would it not be the same for each subject? Why do we perceive the same object in different ways depending on age, emotional status, physical conditions, and the like? How can the physical world, albeit reconceived in terms of relative properties, cope with the familiar subjective variability of experience?

Positively, object-relativity explains away subjective variability. The short answer is that since the object's properties are relative to our body, each difference in our body will determine a difference in the properties of the object. If the world is a bundle of relative properties that are what they are because of their relations with other physical objects (in our case, our bodies), each object will have multiple properties—e.g., multiple velocities, multiple colors, multiple shapes, and so forth. In practice, each object exists in multiple versions of itself, each relative to a given object (which is occasionally a human body). Relative objects endorse a much richer physical world than the layman has ever suspected. In such a world the same room is at once hot and cold. It is hot relative to my body after a run and cold relative to yours when you have a fever. The same canvas is at once both white and cyan. The same screen is both white and multicolored. The same particle is both still and moving. The same object appears different to different subjects because the same object is different relative to their different bodies.

The physical object, once it is revisited in terms of relative properties, has as many relative properties as are needed in relation to as many external objects (some of which are human bodies) as are needed. This is not a problem. The object is not overburdened by *ad hoc* properties. If the object is not coupled with another object, certain properties are not pulled into existence. The object has umpteen relative properties that it sustains just in case—“the case” being the circumstances offered by other objects—in this instance, our body. Analogously, a body has as many relative velocities as they are needed given other objects and reference frames.

The relative object can also address the issue of perspectivalness. The properties that constitute each object are relative in nature, and so they will also be perspectival insofar as they are all centered around the object relative to which they take place. The notion of perspectivalness, often quoted as something that is not shared by the physical world *per se*, is part of the very fabric of relative physical reality. All physical properties are relative to a given object and thus they can embody a viewpoint. Consider velocity again. Is a velocity field not centered on and determined by a reference frame? Of course, it is, and the same can be said for all other properties. Every object defines the surrounding world in function of itself as its relative fulcrum of existence. The resulting relative world is perspectival with respect to such an object. The fact that we perceive a world perspectivally centered around our body is not the consequence of the structure of our mental space, but the consequence of a world that exists relative to our body.

If the world is made of relative properties, once again, a puzzled reader may wonder if physical objects have an existence independent of being an object of experience. And would this not be a form of panpsychism? Not at all! I propose that the properties of experience are nothing but physical relative properties instantiated by an object relative to another object (which in our case is the body).

In sum, the richness of a relative physical world—or at least of a world where all properties are relative—explains subjectivity and perspectivalness away. There is no longer any need of a layer of multiple subjective appearances to justify the different ways in which an object manifests itself to different observers. If an object is made of relative properties, given the fact that different observers have different bodies, the same object will have distinct and different relative properties for each of them.

## Allegedly Irreducible Mental Features

What about those properties, such as intentionality and phenomenal character, that an influential philosophical tradition has attributed to the mind (Brentano, 1874; Nagel, 1974)? To address this issue, I distinguish between the properties that our experiences show—as the red of the apple or perspectivalness—and the properties that have been historically introduced to back up the notion of a conscious mind separate from the physical world—as intentionality and phenomenal character. The former group can be addressed by the notion of the relative object we have outlined above. The apple on the table is red, round and shiny just like my experience of the apple. I can therefore argue for their identity. However, the latter group seem different. How can intentionality and phenomenal character be found in the physical world? One may wonder whether such properties, which have been traditionally attributed to mental states, cannot be found in physical objects. I contend that they cannot be found because they are only philosophical inventions. Revealingly, they cannot be found in one's experience either. They do not exist at all—they are not aspects of experience we encounter in our life. They are conceptual crutches introduced to safeguard old prejudices about the mind. In fact, once one adopts the relative object, intentionality and phenomenal character are no longer needed. They are revealed for what they have always been, namely *ad hoc* hypotheses introduced to back up the separation between subject and object. They cannot be found because they exist neither in the physical world nor in one's experience.

### Intentionality/Aboutness

First, consider *intentionality*. Ever since Brentano, intentionality has been considered the mark of the mental. A common view is that mental states have intentionality (or aboutness), while physical entities do not. Pertinently intentionality has never been found, rather it has been postulated as a magic power that would allow the mind to be about something else, a capacity not shared by physical entities. While Brentano's dualism allowed him to appeal to the alleged special powers of the mental, contemporary physicalists do not have such a freedom. In fact, the insistence on intentionality has led to the ever-popular but hopelessly misguided genre of “naturalizing intentionality”—i.e., the recurrent desperate attempts to find intentionality in a physical world that is assumed to be devoid of it—which many philosophers have criticized (Dennett, 1987).

Experientially, intentionality has always been immaterial. Humans beings have never experienced it. Philosophers have postulated it. Intentionality has been a philosophical invention hinged on the assumption that we are not the world we perceive and thus that we need a metaphysical arrow to cross the gap between us and the world. Yet, when we see a red apple, we do not experience our mental states in their intentional relation with the apple. We perceive the apple. There is only the apple, as suggested by the transparency of perception (Harman, 1990; Martin, 2002; Tye, 2010). When we experience the world, the world presents itself to us. If intentionality were real, it would not only be physically untenable, but also phenomenologically immaterial and altogether flimsy. There is no intentional coat over the world as we experience it.

Object-relativity allows us to set aside intentionality by embracing an identity between the mental and the physical. However, not in the sense that the mental is identical with neural processes distinct from the object one perceives, but in the more radical way that the mental is identical with the physical object itself. From this perspective there is no longer any need for the mental *to be about* something, because the mental *is* the thing itself the mental has been requested to be about. Once identity is revealed, aboutness is no longer needed. My experience is no longer *about* the apple. My experience of the red apple *is* the red apple. My neural activity does not need to be about anything. When I perceive the apple, I don't do it in virtue of a mysterious (and invisible) intentional relation between my neural processes and the apple. I perceive the apple because, at that moment, the thing that is one and the same with my conscious experience is the apple itself. Perceiving is being. What is my experience of the apple? It is the apple. *Identity is the new intentionality*. Thanks to relative objects, the concept of intentionality/aboutness can finally be retired. Identity is all we need.

### Phenomenal Character

Now consider the second alleged irreducible feature of the mental: its alleged *phenomenal character*. I contend that—as with intentionality—the notion of phenomenal character was introduced to safeguard an over simplistic notion of physical objects. Yet, no one has ever experienced the alleged phenomenal character of experience. It has been another philosophical invention rather than an experiential fact.

In fact, the main reason for keeping phenomenal character apart from the physical world has been that scholars have claimed that experience has a phenomenal character that does not exist in the physical world. Thus, they have claimed that, for example, my experience of red has a phenomenal character that does not exist in strawberries and cars. The main reason for such an argument was that the properties one experiences do not match with the alleged absolute objective properties of physical objects. This was only a big misunderstanding. In fact, it was not understood that properties are relative, one class of properties was selected as the real objective one. All other relative properties were downgraded to be nothing more than subjective appearances. However, I argue that, thanks to the relative nature of the object, the properties we experience can be relocated in the physical world, where we have always experienced them—namely in the external objects. So, the red is not created by neurons, the red is instantiated by the apple relative to a trichromatic visual system (which is yet another object). In this way, I argue, there is no reason to defend the existence of phenomenal character. We do not experience phenomenal properties as opposed to physical properties. In our experience, and world, there is only one red, neither phenomenal nor physics-al (in Strawson's sense of physics-alism). No one has ever seen a phenomenal red next to a physical one. I have never experienced the phenomenal character of red. I have always experienced the red of strawberries and cars. The properties of the physical world are not different from the properties we experience day in and day out. In fact, as far as empirical evidence goes, there is only one class of properties. There is only one red.

Consider colors again. Color scientists often state that colors do not exist in the physical world (Galilei, 1623; Zeki, 1980; Eagleman, 2011). However, how could brains create something that is not part of the physical world? When neuroscientists claim that colors do not exist in the physical world and yet are created by (or exist in) the brain, they contradict themselves. The mystery of how experiences (e.g., of something green) can have the qualities we experience is explained by taking those experiences to be identical to external objects. The mystery stems from looking for the property of being green in the wrong physical place—the brain—where nothing is green. If we had looked in the external objects from the start, the notion of phenomenal character would have never been put forward. Green peas and red apples are more obviously green and red than neural activity can ever hope to be. From the fact that the brain does not have the properties of the apple, many have concluded that phenomenal properties (redness, roundness, and applishness) are not identical with any physical property. This is a logical mistake—inferring that the categories of the physical and the mental are different because the chosen individuals (brains vs. apples) are different. On the contrary, the correct conclusion should have been that the alleged hosting object (the brain) is the wrong one since it does not have any of the properties we experience.

Note how, in everyday life, *the notion of “experience of such objects” is redundant to that of the objects themselves*. Basically, the same entities are counted twice. In real life, we cannot distinguish our reference to an apple from our reference to an experience of an apple. If I want to point to my experience of the apple, I can only point to the apple I hold in my hand. It would not make any sense to point to my head, because there is nothing in my head that looks like my experience of the apple. Experience *qua* experience has been invented to locate the external world where it was not, namely inside our body. As a matter of fact, there are no *a priori* reasons to assume, as Bertrand Russell did with his version of monism (Russell, 1921), that the physical thing—whose properties are the properties of our experience—is our brain. It might be something else whose physical properties might be much more similar to (if not identical with) the properties of our experience. The object is the best candidate. As a candidate for being my experience of the apple,*the apple is much better than the brain*.

In the past, many scholars have maintained and believed that objects are akin to their scientific image, a mathematical wireframe devoid of qualities—a position defined by Galen Strawson as “physics-alism” (Strawson, 2017). But why should we assume that is the case? An object cannot be completely stripped of its properties; an object is how it presents itself to us. The properties we find in our experience must be in the physical world—if we are physicalists, where else should they be? And if they are in the physical world, what place would be better than the object itself? Note that placing such properties in the brain, as neutral monists do, does not simplify the matter. On the contrary, it adds mystery to the puzzle of how the properties of an external object are instantiated by a physical structure—the brain—that does not reveal such properties when directly observed. In contrast, by means of identity with the object, it is possible to reload the whole issue.

The bottom line is that phenomenal and physical properties coincide completely—not in the sense advocated by a panpsychist, which is that phenomenal properties are prodigally distributed everywhere—but in the more parsimonious scenario where physical properties are one and the same with those that we call phenomenal. The phenomenal is nothing but the physical. Between physics and personal experience there is no mismatch, neither conceptually nor phenomenologically. In everyday life, I see an apple and I grab it. Where is the mystery? The properties of my experience are the properties of the apple—what else should they be? My experience and my world are one and the same.

## Hallucinations and Dreams

If Jacqueline's experience of the apple is the apple, how can cases in which there is no apple and yet Jacqueline experiences an apple be explained? This is the traditional argument from hallucination: S experiences O but there is no O (Macpherson and Platchias, 2013; Phillips, 2013; Genone, 2014). In fact, all forms of hallucinations and of misperceptions challenge realist models of experience because they suggest that what we experience does not occasionally exist, which seems to rule out the possibility that our experience is either identical to or constituted by external objects. The so-called common kind assumption (as stated in & Haddock and Macpherson, 2008) has often been used to endorse indirect perception—which is why many realists have rejected it and have embraced disjunctivism (Byrne and Logue, 2009). Consequently, the view presented here—based on the identity between the external object and one's experience—is an extreme form of realism and thus it must address this key issue.

A first line of defense against this objection is that the empirical evidence, contrary to widespread belief, provides only scant support if any to the notion that the brain produces phenomenal experience endogenously. Yet, many layman's notions—e.g., the brain in a vat hypothesis—have never received any empirical confirmation. In fact, there has never been an actual case of a brain in vat. All empirical cases are based on subjects that became isolated at a certain point in life, such as in the Locked-In Syndrome (Bauer et al., 1979; Laureys et al., 2005). From a philosophical perspective, these cases are not valid because such subjects have had previous physical contact with the physical world. They are not cases of brains in a vat.

Second, to the best of our knowledge, all cases of phenomenal experience can be traced back to an actual encounter with a real physical property. Although many philosophers and scientists have been keen to attribute to the brain the capacity to generate new phenomenal experiences—e.g., Hume's missing shade of blue—the available empirical evidence does not support this claim. For instance, born blind subjects, contrary to common beliefs, have never reported any color in their dreams or during hallucinatory states (Kiekopf, 1968; Raz et al., 2005; Amedi et al., 2010). Such subjects have reported shapes and other physical properties that they explore using tactile modes of exploration. Anecdotal cases of innate mental visual imagery are rare, anecdotical, and questionable (Kennedy and Juricevic, 2006). Likewise, direct brain stimulation does not lead to arbitrary novel elementary experiences but triggers new combinations of previous moments in one's life (Penfield and Rasmussen, 1950; Penfield and Perot, 1963; Lamme, 2006; Ptito et al., 2008). Experimental cases in which one's experience is possibly (yet not surely) extended to novel features have always required prostheses or other contraptions that might effectively allow a human body to single out new external physical properties (Crane and Piantanida, 1983; Hsieh and Tse, 2006). Consistently, dream reports do not indicate radical departures from one's everyday life (Domhoff, 2001; Cicogna et al., 2007; Domhoff and Schneider, 2008; Bulkeley, 2009). Our dreams are populated by the same colors, tastes, smells, shapes, sounds, and properties we encounter round the clock (Revonsuo and Salmivalli, 1995; Hurovitz et al., 1999; Kerr and Domhoff, 2004; Schwitzgebel et al., 2006). Of course, in dreams and hallucinations the combinations in which we perceive such elements are different from those in which we have encountered them. Similarly, Charles Bonnett patients' experience is made of reshuffled combinations of their life (Teunisse et al., 1996; Ffytche, 2005; Hedges, 2007). The bottom line is that our brains do not seem capable of creating new elementary principles. Remarkably, Descartes himself recognized that imagination and dreams are unable to concoct truly novel stuff, only to rearrange previous impressions (Descartes, 1642):

How often has it happened to me that in the night I dreamt that I found myself in this particular place, that I was dressed and seated near the fire, whilst in reality I was lying undressed in bed! For, as a matter of fact, painters, even when they study with the greatest skill to represent sirens and satyrs by forms the most strange and extraordinary, cannot give them natures which are entirely new, but merely make a certain medley of the members of different animals.

Thus, dreams and imaginary constructs are like painters who need to patch together existing animal body parts and colors. So much the worse for the alleged power of the mind to create content! Creativity is more like a process of recombination of the external world than a process of creation of elementary components. We cannot create a new color hue out of sheer imagination. Picasso famously stated “I don't create. I find.” Mental creativity is akin to the creation of new species out of evolutionary processes that reshuffle our DNA and obtain new organisms. This is consistent with an empirical stance about creativity. Creativity is not a mystical access to a world of new pristine platonic ideas, it is a natural process of recombination of existing stuff.

Empirical reports and phenomenological accounts are amenable of endorsing a daring hypothesis—namely that all cases of experience are a recombination of actual physical features we have previously encountered. Mental features are never concocted out of thin air. As the chimera is a hybrid creature composed of the parts of a lion, a goat and a snake, so our dreams and hallucinations are *chimeric* in the sense that they are composite amalgams of existing external physical properties and events.

If perception is explained by an identity between the external object and one's experience (rather than by an identity between a brain process and one's experience), in principle nothing prevents us from considering the possibility that whenever we experience something—say when one dreams of a pink elephant—one is perceiving previous events. Previous instances of pink and elephants are the recombination that is perceived as one object. In fact, they are one object, albeit unusual. In hallucinations, one perceives a physical object that is a reshuffled combination of previously encountered objects.

However, the objection still stands, when you dream of an apple today, but the apple was eaten yesterday, the apple is no longer there at the time of the dream! Thus, in such a case, it may seem that the apple cannot exist at the time of your dream. To solve this obstacle, we need to reconsider further the notion of the now (the present). There is always a greater-than-zero time span between the external object and one's brain activity in standard perception. We cannot catch flies, because they move so fast that they are always elsewhere than where we have just seen them. It is a scientific fact that standard successful perception is never synchronous with the external object. In regard to neural activity, the world we live in is always in the past and there is no fixed threshold as to by how much. When we look at the moon or the sun or the stars, the object that is identical with our experience is seconds, minutes, or years in the past, sometimes well before our body even existed. Crucially, while such objects are in the past of our body, they are in the present of our experience, which is identical to the objects rather than the body. Mistakenly, in the case of standard perception, the object we perceive is taken to be there and now, at the time and the place where our body is. Yet it is not so. The picture is a gross approximation and a physical mistake. The object we perceive is never in the same place and time where and when the corresponding brain activity occurs. It might be a few inches or thousands of miles far off. I can look at an apple on my desk or I can look at the sun or even at a faraway star. From a spatial perspective, the notion that the object is “there” can be arbitrarily stretched. “There” can mean something as far away as billions of miles.

Here one might object that: “The star relative to my body is light years away, and appears so, so my experience is identical to an object several light years away. But the star is not several light years away from itself, so my experience of the star is not identical with the star itself.” Note the mistake in assuming that being away from the body means being away from the self. The objection reads “my experience is identical to an object several light years away.” Away from what? Of course, it is implicitly assumed that the object is away from me, but it is not. It is away from my body, which is the thing that I am not—unless my body is the object of my perception as is the case when I look at my hands or I focus my attention on my heartbeat.

Once the notion of “there” has been stretched *spatially*, one might venture to stretch the notion of “now” *temporally*. In fact, every phenomenon extended in space is also extended in time. Thus, although most everyday phenomena are so close that the time delay is negligible, they are not synchronous either. In many cases, the time delay is relatively long. A familiar object like the moon is one second away from the corresponding neural activity; the sun is 8 min away; a star can be years away. And yet, they are far away from our body, not from us. They are inside our present. They are our present.

As we have seen, due to nomological speed limits, everything we perceive is—to some extent—in the past. The present is not what takes place at a given time, but rather is the set of events that causes neural activity at a given time. The present is what is *causally present* to my body rather than what takes place at the same time in which neural activity does. Thus, *the star is as present as the apple*, notwithstanding the different time span of the involved causal process. In memory, dreams, and hallucinations, past events are still causally present. They are as physically present as objects are in standard perception. The apple your experience is identical with it is not the apple at the time of your neural activity (there is no longer any apple), but it is the apple at the time your body met it (yesterday).

In short, my approach to dreams and hallucinations is straightforward—whenever S experiences O, O is present, provided that the notion of present (there and now) had been revised and stretched in causal and relative terms as suggested by contemporary physics. So dreams and hallucinations can be recast in terms of reshuffled perception of spatiotemporally spread physical objects. Therefore, they can be explained as cases of identity with spatiotemporally spread physical objects.

Whenever, we experience something—be it a standard perception, a dream, or a hallucination—to the extent that what we perceive has occurred at some place and time, the object is still the cause of one's neural activity. Thus, the object may well be the thing that is identical with one's experience, no matter when and where its parts occurred.

**Figure 1:**
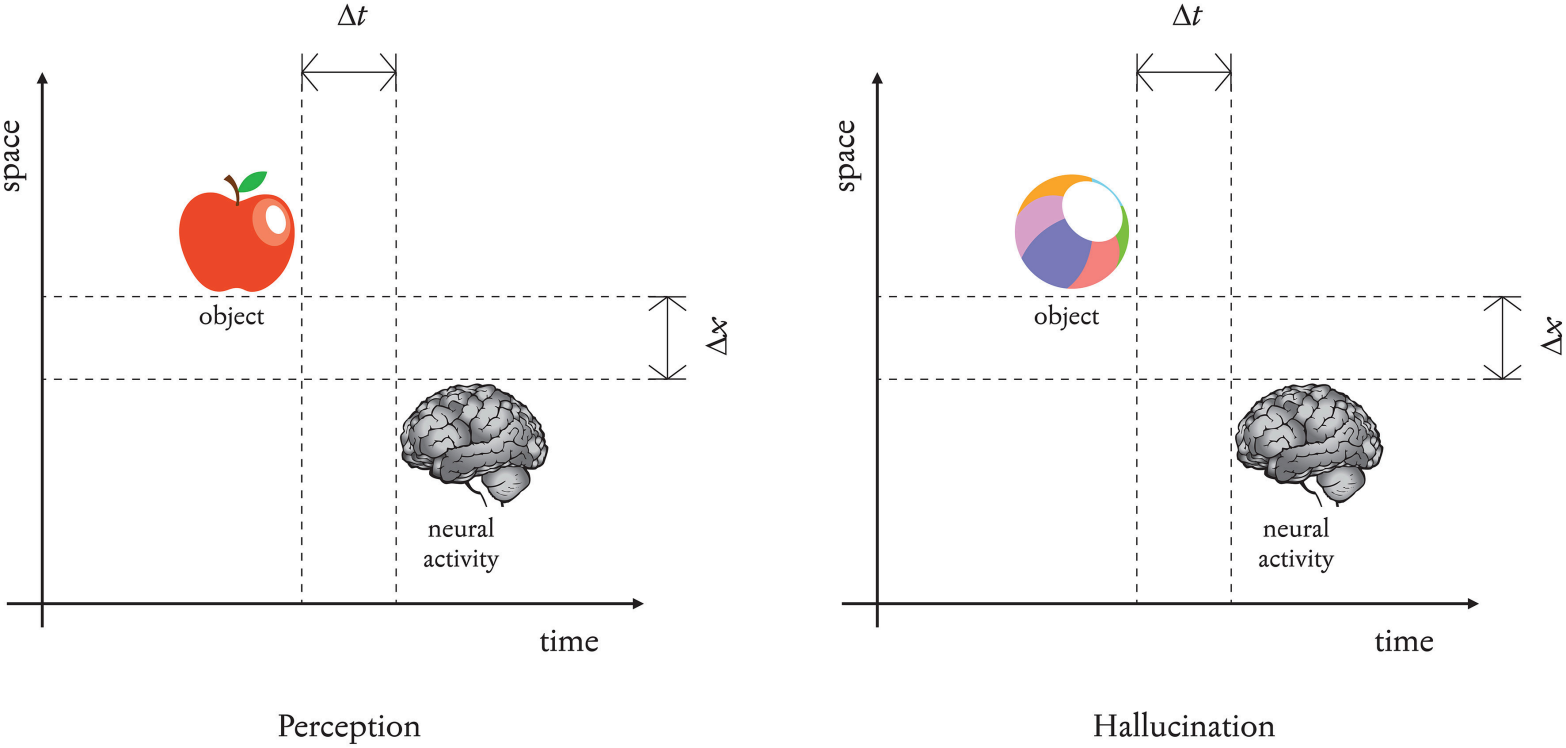
Perception vs. hallucination. They are the same. Only the length of the time span is different.

In comparison, the standard view—albeit apparently more reassuring—is crammed with problems. In fact, the notion that one perceives only *nearby* or *proximal* objects is parochial and vague. How near should an object be to be near enough? There are not known valid thresholds. All objects and events occur previously to one's neural activity. Because of this gap, the time-lag problem kicks in. By nomological necessity, the external cause of everything that takes place in our brain is always in its past (Δ*t* > 0). Such a past can be relatively near or very remote, as is the case with astronomical objects, but it is past nonetheless. *Perception is never instantaneous*. Any object is always at the beginning of a process spread over a time span and across a spatial extension. A “close enough” boundary is an empty notion. Thus, we can turn the traditional time-gap argument upside down. If stars and long-gone events are rejected as the causes of one's memories, dreams, and hallucinations, by the same token all everyday objects ought to be rejected. Such an argument, if valid, would limit one's world to the very proximal shell that surrounds one's skin and receptors, which is absurd. In fact, we do not perceive a thin layer of events enveloping our bodies. We perceive external objects wherever and whenever they are. Our experience is identical to such objects whenever and wherever they are. So, the time-gap argument may be used to show that hallucinations and dreams are forms of perception (Figure 1).

To recap, traditionally, the argument from hallucination is based on two premises, namely: (1) S experiences O; and (2) O does not exist. Here I argue that the former is true, while the latter is not. Whenever S experiences O, O does exist! However, O exists where and when it likes, so to speak.

For instance, take the pink building I see now, the elephant I will see tomorrow at the zoo, and a flying bird I will see next week—all together, these objects will produce an effect in my brain 1 year from now, for example the night of May 15 2019. On that night, sleep-related mechanisms will shield my body from the surroundings (for example shutting down the thalamic channels). As a result, those three events will cross their causal paths and produce a joint effect. They will be causally present to my brain insofar as my brain will offer them the proper circumstances for causing an effect together. Their joint effect will carve out their cause, which is bizarre-yet-physical whole composed of three aspects: pink + flying + elephant = a pink flying elephant. I will then dream of a pink flying elephant, and yet the pink flying elephant will be physical, like my son's face looking at me right now. Emilio's face is composed of two eyes, a nose, and a mouth. These are three separate objects that, because of my body in front of him and thanks to my fusiform gyrus, produce a joint effect and are brought into existence. Likewise, the pink flying elephant is made of three separate objects that, because of my body in that future bed, will produce a joint effect and will thus be brought into existence as a composite object. In standard perception objects are very often composed of parts taking place roughly at the same time. An exception is represented by music where we perceive tunes made of notes occurring at different times. In dreams and hallucinations, we are identical with objects made of parts scattered in space and time.

To recap, during wakefulness, a series of mechanisms guarantee that one's world is made of only the relative proximal objects. This is useful and efficient. Normally, one is coupled with the most pressing parts of one's environment. In various circumstances, though, one's world is made of combinations of objects belonging to the whole causal story of one's body. Such combinations are just as real and physical as the more proximal objects. While they may be less useful, such combinations are as real as the former. Thus, hallucination is perception of causally reshuffled actual objects. Hallucination is perception. Perception is experience. Experience is identity.

One might wonder whether the claim that hallucination and perception are the same is not too radical. In fact, the prevailing stance in neuroscience is the same, namely that perception is like hallucination. Most neuroscientists and philosophers of mind—e.g., Koch, Tononi, Seth, Eagleman, Revonsuo, Zeki—have claimed that perception is a form of reliable hallucination. They have claimed that the world we see is a mental representation created by the brain. Thus, it would be unfair to challenge the presented proposal on this issue and not to do the same to such views. Crucially, these neuroscientists claim that perception is like hallucination, while the Spread Mind claims that hallucination is like perception.

As to the relevance for psychology, I'd like to add that, in this account, hallucination differs from perception on a practical level while being the same on an ontological level. In traditional accounts, hallucinations are mental representations triggered by internal causes and perceptions are mental representations caused by external objects. According to the spread mind, hallucinations are external objects causally connected to our body through relatively unusual pathways, while perceptions are external objects causally connected through familiar pathways. Hallucinations are akin to seeing objects in a kaleidoscope or in a reflection. One sees real objects, only that, because of reflections, one sees them in the wrong place. Yet, one still sees actual objects. Seeing an object in a mirror is not ontologically different from seeing something in standard conditions. In both cases there are light rays connecting the object with our retina.

The good news is that this model is amenable of empirical verification and thus it can be falsified. If one ever hallucinated something whose elementary constituents were not part of our physical relative world, the presented model would fail. So far, though, the empirical evidence points in the opposite direction—everything we experience is made of parts that have been part of the world we have lived in and so it is still causally present relative to our bodies.

## I Am Not My Body

Many objections to the presented theory are likely the offshoot of not taking the key hypothesis at face value, namely that the thing that is our experience—and thus us—is the external object, revisited in terms of relative properties, rather than the body. In fact, while we perceive the location of our body, there is no evidence that we can perceive the location of our experience *per se*—a point already well-taken by Daniel Dennett (Dennett, 1978). In this regard, contrary to popular wisdom, decades of neuroscientific research have unanimously shown that consciousness is not inside the brain. The hypothesis I propose—i.e., experience is identical with an object and thus is different from the body—requires a conceptual jump: we are no longer our body. Departing from our body might not sound like an easy step and both language and layman's prejudices threaten us to relapse into more traditional solutions.

The mind-object identity entails that we are not a body (or a subject) that experiences, we are the objects that exist relative to our bodies. The body does not feel. The body is the physical condition for the existence of the things our experience (us) is identical with. Once we embrace this conceptual shift, many objections will lose their apparent intuitive strength.

A first group of objections is raised around the concerns that our experiences might possibly be far away in space and time from our bodies. A different way to illustrate the same concern is to point out that conscious experience cannot be stretched at huge distances as the proposed theory implies. Some authors would be more comfortable if the experiential present was stretched only over a local portion of space and time. They might find it difficult to accept that the present can extend out to a star that may have ceased to exist by the time light from it hits the perceiver's retina. I have already addressed this issue. Once again, such objections are the outcome of assuming that, whatever we are, our conscious mind is roughly located where our body is. Yet, this is precisely the matter of contention. In fact, my proposal denies explicitly the colocalization of body and experience. Our experience is not where our body is. Our bodies are the condition for the existence of the things that are identical with our experiences, no matter where and at what time they are located. Bodies are not the place where our experience is.

Many readers move from the commonsensical notion that the physical basis of consciousness is located in a relatively small spatiotemporal area centered around the brain, roughly a few centimeters large and a few hundreds of milliseconds long. Yet, such a commonsensical notion is based on two implicit unwarranted assumptions: (1) that consciousness is located inside the head (which is precisely the premise my theory calls into question and which is something that has to be proved rather than assumed), (2) that the physical processes leading to conscious experience must be comparable in size and time to the processes taking place in a human body (digestion, metabolism, and so forth). Both assumptions are unsupported by evidence. In fact, why should it be so? Is there any physical law that dictates that physical phenomena have only a limited spatial length and time span? No. Is there any proof that we are inside our brain? No. The notion of a “local portion of space and time” can appear familiar and reassuring, but one may still ask “local to what?” Implicitly, the notion of being local implies to be close to the supposed locus of one's mind. But this makes sense only if it is assumed beforehand that such a locus is inside the brain. Conceptually, if consciousness is relocated where the object is—because the experience is identical with such an object—there will be no more distance between “us” and the “external object.” Our experience, which is us, will occur where the object is—the two being identical. So, there is no reason to deny placing experience far away from the body. There will be no distance whatsoever between such objects and us. We are “there” where the object is, rather than “here” where the body is. So, if my experience is a relative physical property instantiated by the star relative to my body, my experience will not be light years away from me, it will be light years away from my body—the far away stars and my experience being the same. Clearly, for the layman “here” does not refer to the location where my experience is, but to the location where my body is.

As to whether many years and many light years should be less acceptable than a few cm and fractions of seconds, as it is routinely accepted in neuroscience, one can only point out that the difference is a parochial notion unsupported by any physical evidence. Between the far away star and our eyes there is a photon. Between the apple on the table and our eyes there is a photon too. What is the difference? The length of the photon? Surely nobody will seriously argue that short photons are fine and long ones are not! Likewise, the idea that there is a timeframe in which current experiences happen is parochial. The timeframe in which an effect takes place in our body (as the outcome of a causal chain) is not the timeframe of the cause that is identical with one's experience. Neural processes take place in a time-limited timeframe, but such a timeframe refers only to the last part of a longer physical process, whose onset (and not its end) is the physical entity identical to our experience. we're the cause, not the effect.

Another related group of objections is centered about the notion of agency, a huge topic I can hope only to mention here. One might worry that if our experiences were nothing but the (more or less far away) objects, we would be acted by the objects themselves. The short answer is no: we are not acted by the objects, we are identical to them. As aforementioned, we are the cause rather than the effects. A longer answer would require one to dig deeper into the causal structure between external relative objects and bodies. So, for example, while my experience of the star may cause me to sing, because of the proposed identity, the star itself is the cause of my singing. One may be puzzled by this statement since one may assume that causes require proximity, and thus the photons that hit my retina cause the experience that cause the singing. Yet, why should only proximal causes be the relevant ones? In our everyday life, we do not apply the notion of causal proximity. Otherwise perfectly reasonable statements such as “The asteroid that hits the earth 75 million years ago caused the development of mammals” or “Smoking from 1950 to 1990 caused my aunt to die of emphysema in 2004” would be rendered meaningless. Reasoning in terms of causal proximity would have nefarious consequences for internalism too—our actions would be acted by the very last neural activity in the chain, which is equally absurd.

However, it might be objected that the problem of identifying experience with the object itself is that we want to be the case that our experiences cause things, e.g., Jack salivates upon seeing a juicy apple. The reply is that Jack's body salivates, not Jack. Jack is the cause that makes Jack's body salivate. Jack's experience is the apple that Jack's body brings into existence. What's the cause of Jack's body salivating? The apple. Thus, strange as it may seem, it is the apple that acts through Jack's body, which is consistent with the claim that objects are one's experience. Since the apple is Jack, everything adds up. It's Jack that make Jack's body salivate. So the identity between objects and experiences is consistent with experiences being the cause of one's actions.

Of course, I do not mean that such relative objects (or objects with relative properties) have experiences. I do not mean that, since the experience of the apple is the apple that exists relative to my body, then the apple experiences. That would be both crazy and question begging. Objects do not have experiences, rather our experiences are identical to the properties of the external object relative to our body. The proposed account maintains there is no such a thing as “S experiencing O.” The proposal is that “S experiencing O” is a way to refer to the fact that *O exists relative to S's body*. Here, experience is revisited in terms of physical existence, which is what should be expected. In passim, traditional mind-brain identity theories aimed to do so. So, the proposal is that the physical thing one calls “my experience of the apple” is “the apple that exists relative to one's body.” There is no longer the need to postulate a subject—be it the brain, the subject, or an immaterial self—who experiences the world. There are only lumps of objects that exist relative to bodies, or relative to other objects. Yet, bodies do not experience. Brains do not feel. Eyes do not see. In fact, nothing experiences, feels, or sees. Things exist though. Things exist relative to bodies, brains, and eyes. Ontology is phenomenology as it should always have been in a physical world. The existence of such relative physical objects is identical to what has been called the experience of the same objects. Experience is relative existence.

One may object that it is not any easier to claim that there is an identity between object properties and experience than between brain processes and experience. Yet, this impression may result from assuming that the properties of the object are of absolute nature rather than being relative properties carved out by the causal relation with one's body.

While the former do not coincide with our experience, the latter are just what our experience is. One may worry that the surface of the apple is not like my experience of the apple, yet the surface is not a relative property of the apple. The surface is the alleged objective feature of the apple, which is independent of the visual system with which it interacts. The surface as it takes place relative to the visual system of a trichromat is a different entity/property and it is just the kind of thing we find in our experience. Or so I claim.

As to the latter part, I want to stress once again the difference between physical properties as something that is absolute and physical properties as something that is relative to one's body. While the former does not coincide with our experience, I claim that the latter is just what our experience is. I mean, the surface is not the relative property of the apple. The surface is the alleged objective feature of the apple, which is independent of the visual system with which it interacts. The surface as it takes place relative to the visual system of a trichromat is a different entity/property and it is just the kind of thing we find in our experience. Or so I claim.

Likewise, we do not need to smear the relative object over the whole causal process, because relative objects do not have to reach the body as the magic gate to one's consciousness. The idea that the external object must reach the body is—once again—based on the idea that we are located where our body is. On the contrary, the body is the fulcrum of a causal chain, whose cause is external to it. So, while photons are not red, and neither are apples+photons+eyes+brain, there must be something that is red, otherwise what will we see? The red I see there is the relative property that the apple instantiates relative to my body. The red is the cause of a neural process that provides the condition for the existence of that specific property—no matter how long and complex the causal chain is. If the red thing is Mars, so much the better.

Likewise, one might disagree that an object does not instantiate a given property until such a property becomes the target of one's experience. Not exactly. Rather, an object does not have a given property until the conditions for the existence of such property are met. For instance, would an isolate particle have any velocity if there were no other particles around? Of course not. Would it make any sense to ask what the velocity of an particle is before another particle enters the scene? Of course not. There was no velocity in particle A until particle B was included. The same holds true for any other property. Consider color. Imagine a red ruby on a planet many light years away from earth, on which there are neither humans nor alien trichromat observers. What could we say about the color of the ruby? Does it have any color waiting to be seen? Of course not. Color exists only relative to the right physical system, which is missing on that planet. At some point, a trichromat is born on earth and points his telescope toward this distant planet and observes the ruby. At that moment, for the first time, the color property of the ruby takes place actually and relatively to the body of the trichromat at the other end of the telescope. The ruby has existed light years away and possibly even before the trichromat observer was born. Was it colorless? Yes, just like an isolated particle was velocity-less until considered relative to another particle. When the trichromat is born and focuses his telescope on the planet with the ruby, color takes place. When and where exactly? On the far away planet at the time when the light started its journey, no matter how many years in the past. Red comes into existence when it takes place relative to a proper external object.

One further objection might stem from a confusion between thinking and experiencing. The spread mind is a hypothesis about the nature of our experience—i.e., consciousness or phenomenal experience, it does not address the issue of thinking and cognition. In fact, while thinking may be accompanied by some experience, it is not like having an experience of what thoughts are about. A congenitally blind subject can think about colors without any experience of colors (or, as I would say, any colored experience). One may think of Atlantis only to find out that the lost sunk island was only a literary invention. One may think of the concept of a dinosaur existing millions of years ago, but that does not entail any experience of the dinosaur as it was millions of years ago. One can see a picture of a dinosaur or the skeleton of a dinosaur exhibited in a museum. They are all events taking place in one's environment. In brief, thinking about something and experiencing something are two separate issues. The Spread Mind addresses the latter issue.

Yet, one may still worry that experience may be requested to move from one place in the universe to another at impossible speed. To clarify, what does it happen when one looks up to the sky and is seeing a star located in a distant part of our galaxy (say location A) and then one moves the eyes just slightly and is seeing another star right (location B). But while the starts are right next to each other in one's optic, the stars themselves are light years apart. Does this imply that, as one's experience moves from one star to another, experience has covered several light years in an instant? Of course not. Experience does not move from A to B. Rather experience is made of a star at location A at time t1, and then it is made of another start at location B at time t2. There is no substance corresponding to one's experience moving from point A to point B. The two experiences are bound by having two effects at close temporal and spatial distance in one's brain. What matters, from a physical perspective, is the causal processes going from the start to one's brain and from one brain event to the next. These causal processes do not contradict any physical laws. The case is similar to the difference between causal processes (which are constrained to light speed) and pseudoprocesses (which are not constrained by light speed) as they have been described by Hans Reichenbach (Reichenbach, 1958). He made the examples of a turning powerful spotlight casting its light against a wall at an astronomical distance. In principle, the illuminated area will move at a speed much greater than that of light.

Having said that, one may see the Andromeda galaxy and a car even if they are distant millions of years in time and space because there is a causal connection between them and one's body. Of course, this is not more mysterious than seeing a distant mountain top and a nearby butterfly, which are separate by a few picoseconds and a few kilometers. Causal connections put them all in contact with our body.

A final objection is related to well-known features of experience such as color constancy. Sometimes, what we perceive seems to be constant notwithstanding changes in the external world. This is an interesting case because it suggests a scenario symmetrically opposed to that of subjective variability. In perceptual constancies, the world changes but our perception does not. How can this phenomenon be explained by the proposed identity between external objects and our perception? It is quite easy. To demonstrate, I will use once more an analogy with velocity and spatial orientation in the field of video recording. Imagine having a device that maintains your camera at the same distance from a target. The device is also able to keep your camera stabilized with respect to the target orientation, so that if the target tilts by, say, 30 degrees, the camera does the same. Once a camera is equipped with such a contraption, the relative position of an object stays the same even if the target object is moved. You will see the object always at the same distance and position. This is analogous to what happens in color constancy; our visual system adapts so that the relative property is the same notwithstanding changes in the external environment. Imagine having a receptor that is able to fine tune the spectrum absorption curve so that the relative position of a spectrum in the color space remains the same. The environmental light might change and as would the reflected spectrum, but the relative color spectrum remains the same, just like the relative position and orientation of an object might remain the same. Thus, the fact that our experience is identical with relative properties is compatible with perceptual constancies. The supervenience basis of one's experience is a relative property that is the outcome of both the external conditions and the affected physical system. The latter may change to compensate the former and allow the external object to instantiate the same relative property—be it velocity, orientation or color spectrum.

The issue of perception constancy allows me to briefly address the causal/constitution discussion. What is the supervenience basis of experience? The answer is straightforward: it is the external object as it exists relative to another object (which in our case is the body). So, it is possible that, say, a faraway star and a very modest light source at a shorter distance will instantiate the same relative property and thus they will be the basis for (they will be identical to) the same experience. They will be the same experience because they will be nothing but the same relative property. Identity entails constitution as well as supervenience. Experience is not caused by object, it is identical to them.

## Conclusion

In 1968, David Armstrong began his seminal book, *A Materialist Theory of the Mind*, by asking the question: “What is a man?” His prompt reply was that a man “is a certain sort of material object” (Armstrong, 1968). Yet, one may further ask, what material object? We know that Armstrong and a good many other scientists and philosophers considered a man to be the human body, in particular the central nervous system. This is one of the most deeply entrenched assumptions in the history of philosophy, in science, and in common sense. The layman feels that we are located where our bodies are. If hard-pressed to be more precise, many would point to their head. Yet, such a feeling and the ensuing notion that the body is the seat of the mind might be wrong, completely wrong.

This paper endorses Armstrong's premise that consciousness is physical, but it proceeds in a radically different direction. Here I have defended an identity theory—only that rather than being a mind-brain identity it is a mind-object identity theory. The material object that is a man is no longer the human body but rather the external object that exists thanks to the causal circumstances offered by one's body.

The presented mind-object identity moves from a revision in the way we understand physical objects which is consistent with both empirical evidence and contemporary physics. Conscious experience is not extraneous and incongruous to the physical world. I do not try to naturalize consciousness; rather I propose to revisit the notion of material object in such a way that experience can be found inside the physical world.

My main claim is that what has been traditionally called “subjective, in the sense of being relative to a subject” can be replaced with “relative, in the sense of being relative to another object.” In the case of human beings, our body provides the required object relative to which the world instantiates the properties we are familiar with.

Previous naturalistic frameworks could not explain conscious experience which was assumed to be extraneous and to some extent alien to the natural order, thus spawning a progeny of chasms: subject vs. object, mental vs. physical, mind vs. nature. While such ontological chasms may be flattering for narcissist subjects keen to be set apart from the world (Freud, 1920), they are ontologically suspicious. To find a solution, the proposed theory does not move from the subject side, but rather from the object side. It does not try to stretch the physical world over consciousness, as other approaches have tried, notably some form of panpsychism, neutral monism, integrated information, and emergentism. On the contrary, the proposed approach moves from two basic notions of contemporary physics. On the one hand, physical properties are relative to other physical systems that offer them the opportunity to take place. On the other hand, everything takes place in a greater-than-zero time span. These two fundamental points unfold a view of nature in which objects are no longer individuals with absolute properties; rather they are rich bundles of relative properties spread in space and time. Such a view, to stress the point again, is not biased by any need of naturalizing consciousness, rather it is a direct consequence of contemporary standard physics. By adopting it, one can put forward the key hypothesis of this paper, namely mind-object identity.

In conclusion, when I perceive a red apple, what, where and when is my experience of the apple? What is the least expensive ontological candidate? My answer is that my experience is identical with the external physical object that exists relative to my body. The relative object is an ideal candidate for identity with one's experience. Nothing is ontologically closer to our experience of the world than the objects the world is made of. Once we set aside the ancient prejudices that our minds are roughly where our bodies are, it is quite straightforward to see that consciousness is—and has always been—there, all around our body, *hidden in plain sight*.

## Author Contributions

The author confirms being the sole contributor of this work and has approved it for publication.

### Conflict of Interest Statement

The author declares that the research was conducted in the absence of any commercial or financial relationships that could be construed as a potential conflict of interest. The reviewer SB and handling editor declared their shared affiliation at the time of review.
